# Rice WRKY11 Plays a Role in Pathogen Defense and Drought Tolerance

**DOI:** 10.1186/s12284-018-0199-0

**Published:** 2018-01-12

**Authors:** Heyoung Lee, Jooyoung Cha, Changhyun Choi, Naeyoung Choi, Hyun-So Ji, Sang Ryeol Park, Seungbum Lee, Duk-Ju Hwang

**Affiliations:** National Institute of Agricultural Sciences, Jeonju, 54874 Republic of Korea

**Keywords:** *Os*WRKY11, Disease resistance, Drought tolerance, Cross-talk, Rice

## Abstract

**Background:**

Plants are frequently subjected to abiotic and biotic stresses, and WRKY proteins play a pivotal role in the response to such stress. *Os*WRKY11 is induced by pathogens, drought, and heat, suggesting a function in biotic and abiotic stress responses.

**Results:**

This study identified *Os*WRKY11, a member of WRKY group IIc. It is a transcriptional activator that localized to the nucleus. Ectopic expression of *OsWRKY11* resulted in enhanced resistance to a bacterial pathogen, *Xanthomonas oryzae* pv. *oryzae*; resistance was compromised in transgenic lines under-expressing *OsWRKY11*. Ectopic expression of *OsWRKY11* resulted in constitutive expression of defense-associated genes, whereas knock-down (kd) of *OsWRKY11* reduced expression of defense-associated genes during pathogen attack, suggesting that *OsWRKY11* activates defense responses. *Os*WRKY11 bound directly to the promoter of *CHITINASE 2*, a gene associated with defense, and activated its transcription. In addition, ectopic expression of *OsWRKY11* enhanced tolerance to drought stress and induced constitutive expression of drought-responsive genes. Induction of drought-responsive genes was compromised in *OsWRKY11-*kd plants. *Os*WRKY11 also bound directly to the promoter of a drought-responsive gene, *RAB21*, activating its transcription. In addition, *Os*WRKY11 protein levels were controlled by the ubiquitin-proteasome system.

**Conclusion:**

*Os*WRKY11 integrates plant responses to pathogens and abiotic stresses by positively modulating the expression of biotic and abiotic stress-related genes.

**Electronic supplementary material:**

The online version of this article (10.1186/s12284-018-0199-0) contains supplementary material, which is available to authorized users.

## Background

Plants in the field are often subjected to abiotic and biotic stresses simultaneously or successively and have therefore evolved elegant mechanisms to respond precisely to individual or combined stresses. Cross-talk between individual stress response signaling pathways is well documented (reviewed in Sharma et al. 2013; Kissoudis et al. 2014; Takatsuji 2017), and evidence has accumulated that the signaling pathways involved in biotic and abiotic stress responses interact either antagonistically or synergistically via the stress hormones salicylic acid, ethylene, jasmonic acid, and abscisic acid (Sharma et al. 2013; Kissoudis et al. 2014; Takatsuji 2017). In rice, many genes are involved at the points of intersection of abiotic and biotic stress signaling, including *OsMPK5*, *OsMPK6*, *OsEIN2*, and transcription factors (TF)s (Xiong and Yang, 2003; Sharma et al. 2013; Ueno et al., 2015).

Bioinformatics and functional analyses demonstrated that TFs, including NAC, MYB, AP2/ERF, and WRKY, are involved in biotic and abiotic stress responses (Atkinson and Urwin, 2012; Shaik and Ramakrishna, 2014). Of these, WRKY TFs are involved in various biological processes, including growth and development (Han et al. 2014; Dai et al. 2016), abiotic stress responses (Kim et al. 2016; Raineri et al. 2015; Yokotani et al. 2013), and biotic stress responses (Abbruscato et al. 2012; Choi et al. 2015; Han et al. 2013; Hwang et al. 2016; Lan et al. 2013; Shimono et al. 2007; Wang et al. 2015; Zhang et al. 2008). WRKY TFs are therefore likely candidates for proteins involved in the cross-talk between abiotic and biotic stresses in rice. Some of these proteins play opposing roles, either positive or negative, during biotic and abiotic stress responses. Involvement of *OsWRKY13* gene in cross-talk between disease resistance and abiotic stress tolerance pathways has been intensively studied. *Os*WRKY13 enhances resistance to *Xanthomonas oryzae* pv *oryzae* (*Xoo*) and *Magnaporthe oryzae* and decreases tolerance to cold and salt stresses (Qiu et al. 2007, 2008). More recently, a molecular mechanism has been proposed by which the suppression of SNAC1 by *Os*WRKY13 enhances drought tolerance (Xiao et al. 2013). The *Os*WRKY45–2 TF confers broad-spectrum disease resistance in rice but reduces adaptation to salt, cold, and drought stresses (Tao et al. 2009, 2011). OsWRKY45–1 confers rice resistance to *M. oryzae* but reduces resistance to the bacterial pathogens *Xoo* and *Xanthomonas oryzae* pv *oryzicola* (*Xoc*) and to cold and drought stresses (Shimono et al. 2007; Tao et al. 2009, 2011; Goto et al., 2015). Interestingly OsWRKY62, one of the group IIa WRKY TFs, plays a positive role in pathogen defense together with OsWRKY45–1 but plays a negative role in pathogen defense under hypoxia stress by self dimerization (Fukushima et al., 2016). *Os*WRKY76 enhances susceptibility to *M. oryzae* and *Xoo* but increases cold tolerance (Seo et al. 2011; Yokotani et al. 2013).

Some *Os*WRKY TFs play positive roles in biotic and abiotic stresses. *Os*WRKY89 [renamed 104 by the Committee on Gene Symbolization, Nomenclature and Linkage (CGSNL)] enhances resistance to *M. oryzae* and UV-B irradiation (Rice WRKY working group, 2012; Wang et al. 2007). It is reported that over-expression of *Os*WRKY30 increases resistance to fungal pathogens and drought stress by two independent research groups (Peng et al. 2012; Shen et al. 2012). For *Os*WRKY71 two independent reports showed that OsWRKY71 enhances resistance to *Xoo* and tolerance to cold, respectively (Liu et al. 2007; Chujo et al., 2008; Kim et al. 2016).

Although evidence for cross-talk between biotic and abiotic stress responses is rapidly accumulating, the molecular mechanisms remain largely unknown, particularly with respect to positive cross-talk. We describe here the role played by *Os*WRKY11 in positive cross-talk between biotic and abiotic stress responses. *OsWRKY11* is induced by heat, drought, combined heat/drought, and pathogen stresses (Shiroto et al. 2004; Ryu et al. 2006). Ectopic expression of *OsWRKY11* under the control of the heat shock protein (HSP) 101 promoter enhances drought tolerance (Wu et al. 2009). These expression patterns suggest that *Os*WRKY11 is involved in biotic and abiotic stress responses. We analyzed the severity of disease in response to *Xoo*, drought tolerance, and expression of stress-related genes in *OsWRKY11* over-expression (ox) and RNA interference (RNAi) lines, and demonstrated direct binding of *Os*WRKY11 to promoters of both biotic and abiotic stress-related genes.

## Results

### Generation of transgenic plants over-expressing and under-expressing *OsWRKY11*

We were most interested in investigating genes involved in the response to combined stresses such as drought and *Xoo* infection. The nine OsWRKY TFs (OsWRKY7, −10, −11, −30, −32, −67, −70, 83 (renamed 94 by CGSNL), −85 (renamed 96 by CGSNL) are previously reported on their induction upon the infection of an incompatible race of *Xoo* (Ryu et al. 2006). In the current study, we focused on *OsWRKY11* (Os01g43650), whose expression enhances drought tolerance (Wu et al. 2009)*.* More recently we reported that *OsWRKY11* expression is increased in compatible and incompatible interaction but the level of its expression is more pronounced in the incompatible interaction than in the compatible interaction (Choi et al., 2017).

**Fig. 1:**
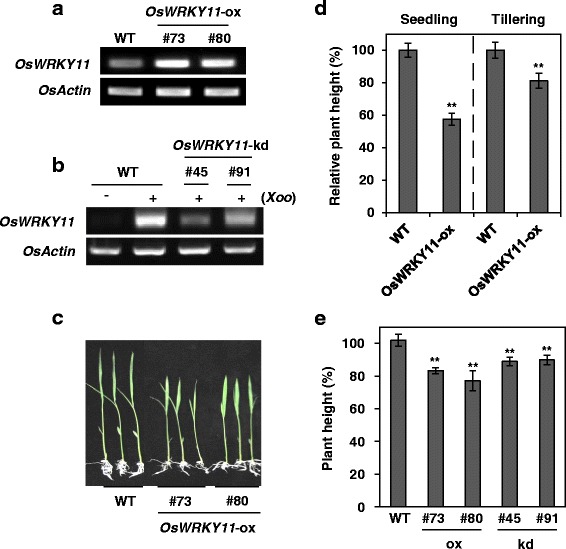
Analysis of *OsWRKY11*-ox and RNAi (kd) plants. *OsWRKY11*-ox lines and *OsWRKY11*-kd lines of Nipponbare cultivar were generated. Over-expression (**a**) and knock-down (**b**) of *OsWRKY11* in these lines were confirmed by RT-PCR with gene-specific primers against *OsWRKY11* (Table S1). Growth retardation of *OsWKRY11*-ox transgenic rice at the seedling and tillering stages. The photograph shows 10-day-old seedlings grown in MS medium(**c**). The relative heights of *OsWKRY11*-ox transgenic plants were obtained by comparing 10-day-old transgenic and WT seedlings grown in MS medium, and 10-week-old transgenic and WT plants at the tillering stage grown in a greenhouse (**d**). Growth retardation of *OsWKRY11*-ox and *OsWRKY11*-kd transgenic rice grown in rice paddy fields (**e**)

To elucidate the function of *Os*WRKY11 in defense signaling, we generated *OsWRKY11*-ox lines and *OsWRKY11*-knock-down (kd) lines by RNA interference approach in the rice cultivar, Nipponbare. We confirmed that *OsWRKY11* was expressed at higher levels in transgenic lines #73 and #80 than in non-transgenic wild-type (WT) control plants (Fig. 1a; Additional file 1: Figure S2), and was compromised in *OsWRKY11*-kd lines #45 and #91 during pathogen attack (Fig. 1b; Additional file 1: Figure S2). Wu et al. (2009) reported that the heights of *OsWRKY11*-ox plants were similar to those of WT plants. Our results, however, were not consistent with this previous report. The heights of the *OsWRKY11*-ox plants, generated in this study and grown in a culture box (Fig. 1c) and a greenhouse (Fig. 1d), were about 60% those of the controls at the seedling stage, but had reached 80% of the controls’ height at the tillering stage (Fig. 1d). As plant height is an important agronomic trait, we examined heights of *OsWRKY11*-ox and *OsWRKY11*-kd plants grown in paddy fields. The heights of *OsWRKY11*-ox and *OsWRKY11*-kd plants were approximately 80% of the height of control plants under these conditions (Fig. 1e).

### OsWRKY11 reduces the susceptibility to a bacterial pathogen, Xoo

**Fig. 2:**
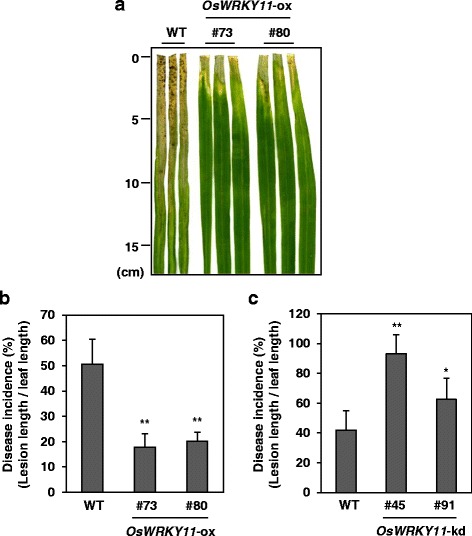
Analysis of *OsWRKY11*-ox and RNAi (kd) plants in response to *Xoo* infection. Wild-type (WT) and transgenic lines (T2) over-expressing or under-expressing *OsWRKY11* were challenged with *Xoo* using the leaf-clip method and photographed at 14 dpi (**a**). Lesion lengths were measured at 14 dpi, and disease incidences for *OsWRKY11*-ox (**b**) and *OsWRKY11*-kd lines (**c**) were expressed as the percentage of lesion length/leaf length. Asterisks indicate significant differences from the WT (**: *P* < 0.01; *: *P* < 0.05)

To examine an effect of OsWRKY11 on bacterial blight disease caused by *Xoo* 10 plants from *OsWRKY11*-ox lines #73 and #80 and *OsWRKY11*-kd lines #45 and #91, respectively, were challenged with a compatible race of *Xoo* using the leaf-clip method. The areas of lesions on the *OsWRKY11*-ox lines #73 and #80 were smaller than those on the WT controls (Fig. 2a, b). By contrast, the areas of lesions on the *OsWRKY11*-kd lines were larger than those on the WT controls (Fig. 1e). This suggests that enhanced expression of *OsWRKY11* results in a reduced susceptibility to the bacterial pathogen *Xoo*.

### OsWRKY11 increases the expression of defense-related genes

**Fig. 3:**
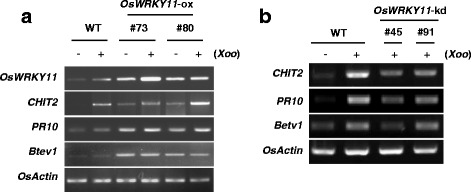
Expression analysis of defense-related genes in *OsWRKY11*-ox and *OsWRKY11*-kd lines. **a** Total RNA was isolated from WT plants and *OsWRKY11*-ox plants either infected with *Xoo* or non-infected. RT-PCR was performed using gene-specific primers for *CHITINASE 2* (*CHIT2*), *PR10*, and *Betv1*. **b** *OsWRKY11*-kd plants were infected with *Xoo* using the toothpick inoculation method, and samples were collected at 24 hpi. Expression patterns of *CHIT2*, *PR10*, and *Betv1* in *OsWRKY11*-kd lines were analyzed using RT-PCR with gene-specific primers. Transcript levels of *OsACTIN* were used as internal controls. These experiments were repeated more than twice

To elucidate the mechanism of *Os*WRKY11-mediated resistance to pathogens, we used RT-PCR to analyze the expression of defense-related genes. Levels of *CHITINASE 2* (*CHIT2*), *PATHOGENESIS-RELATED 10* (*PR10*), and *Betv1* transcripts were higher in the *OsWRKY11*-ox plants than in WT plants before pathogen infection and increased transcript levels of these genes were maintained upon *Xoo* infection (Fig. 3a).

By contrast, induction of defense-associated genes during *Xoo* infection was more highly compromised in *OsWRKY11*-kd line #45 than in line #91. This indicated that *Os*WRKY11 regulated the induction of defense-associated genes such as *CHIT2*, *PR10*, and *Betv1*. Together with the data obtained from *OsWRKY11-*ox lines, these results show that *Os*WRKY11 regulates the expression of defense-related genes, resulting in reduction of disease susceptibility to *Xoo*.

### *OsWRKY11* trans-activates the *CHIT 2* promoter by direct binding in vivo

Since subcellular localization of transcription factor is important to predict its function subcellular localization of *Os*WRKY11 was assessed. 35S::YFP-*Os*WRKY11 was generated and introduced into rice protoplasts along with 35S::NLS-RFP, a nuclear localization marker that contains a classical NLS (Choi et al. 2015). YFP::*Os*WRKY11 was localized exclusively in the nucleus, together with the nuclear marker NLS-RFP (Additional file 1: Figure S1a), suggesting that OsWRKY11 functions in nucleus.

**Fig. 4:**
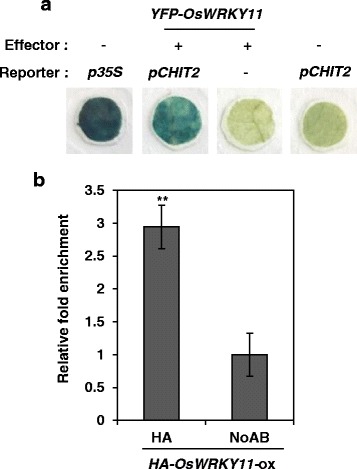
*Os*WRKY11 directly binds to and activates the *CHITINASE 2* promoter. **a** *Nicotiana benthamiana* leaf discs were infiltrated with *Agrobacterium* carrying *pCHIT2::GFP-GUS* alone or with a mixture of *Agrobacterium* carrying *35S::OsWRKY11* and *pCHIT2::GFP-GUS*. Promoter activities in each sample were visualized 2 days post-infiltration using β-glucuronidase (GUS) activity staining. **b** Transgenic rice leaves expressing *35S*::*HA*-*OsWRKY11* were used for chromatin immunoprecipitation with anti-HA antibody. ChIP-PCRs were performed on genomic DNA fragments using promoter-specific primers against *CHIT2* (Table S1). To normalize qPCR values, the value obtained from the sample with pre-immune serum (no antibody) was divided by the value obtained from the sample with 10% input of each sheared chromatin sample and arbitrarily set at 1. The value resulting from *Os*WRKY11 binding to the promoter was expressed as a relative ratio of the pre-immune serum sample. Asterisks indicate significant differences between treatments from the no-antibody control sample (**: *P* < 0.01; *: *P* < 0.05)

Since defense-related genes were highly expressed in *OsWRKY11*-ox lines but their induction by pathogen infection was compromised in *OsWRKY11*-kd lines, we predicted that *Os*WRKY11 was a transcription activator. To test this hypothesis, a yeast vector (BD-OsWRKY11) was generated and transformed into yeast cells. Activity of a reporter gene was assessed (Additional file 1: Figure S1b). We found that *Os*WRKY11 was a transcription activator in yeast. To test this in plants, a promoter-reporter construct containing the *CHIT 2* promoter upstream of a GFP-GUS fusion gene (*pCHIT2::GFP-GUS*) was generated. *Agrobacterium-*mediated transient assays were performed in *N. benthamiana* leaves (Fig. 4a; Additional file 1: Figure S2). Trans-activation activity of *Os*WRKY11 at the *CHIT 2* promoter was assessed using GUS staining. GUS activity was stronger in leaves co-infiltrated with *pCHIT2::GFP-GUS* and *35S::OsWRKY11* than in leaves infiltrated with either *pCHIT2::GFP-GUS* or *35S::OsWRKY11* alone. We also performed a transient assay of promoter activity in rice protoplasts using PEG-mediated transformation (Additional file 1: Figure S3). Luciferase activity was about 2-fold higher in samples co-transformed with *pCHIT2::LUC* and *35S::OsWRKY11* than in those transformed with *pCHIT2::LUC* alone. These results suggest that *Os*WRKY11 trans-activates the *CHIT 2* promoter in plants.

To examine direct binding of *Os*WRKY11 to the *CHIT 2* promoter in vivo, transgenic lines over-expressing HA-OsWRKY11 were generated. In vivo binding of *Os*WRKY11 to the *CHIT 2* promoter was assessed by chromatin immune-precipitation (ChIP) with anti-HA antibody followed by qPCR with primers binding near the W-box or WLE1 (Fig. 4b; Additional file 1: Figure S4). DNA binding was higher in IP samples treated with anti-HA antibody than in no-antibody samples, indicating that *Os*WRKY11 directly binds the *CHIT 2* promoter in vivo.

### *OsWRKY11* positively regulates drought-responsive genes

*OsWRKY11* was initially reported to be induced following infection with pathogens such as *M. grisea* and *Xoo* (Ryu et al. 2006). Later, Wu et al. (2009) reported that over-expression of *OsWRKY11* under the control of a heat-inducible promoter such as *HSP101* conferred enhanced tolerance of heat and drought stresses. Therefore, we performed a drought tolerance assay with the transgenic lines over-expressing *OsWRKY11* under the control of the 35S constitutive promoter (Additional file 1: Figure S5a). Water was withheld from 6-week-old plants for 10 days to induce drought conditions. Transgenic lines #73 and #80, which over-expressed *OsWRKY11*, showed reduced leaf wilting after 8 days of drought treatment compared with WT plants (Additional file 1: Figure S5a). After 5 days of re-watering, *OsWRKY11*-ox plants showed a better recovery from wilting than WT plants. Our results are consistent with a previous report (Wu et al. 2009); however, those authors did not investigate the drought tolerance of *OsWRKY11*-kd lines. We performed a drought tolerance assay of *OsWRKY11*-kd lines by withholding water from 6-week-old plants for 7 days to induce drought conditions (Additional file 1: Figure S5b). After 5 days of re-watering, *OsWRKY11*-kd plants showed a lower recovery from wilting than WT plants. Tolerance to drought was compromised in *OsWRKY11*-kd lines (Additional file 1: Figure S5b). Our kd approach therefore further confirmed the effect of *Os*WRKY11 on drought tolerance.

**Fig. 5:**
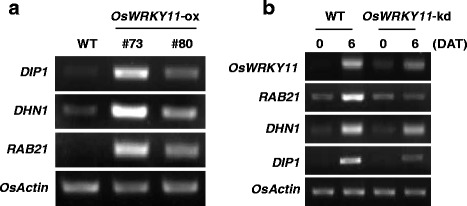
RT-PCR analysis of expression of drought-responsive genes in *OsWRKY11*-ox and *OsWRKY11*-kd plants. **a** Total RNA was isolated from leaves of *OsWRKY11*-ox plants from lines #73 and #80, and from WT controls. RT-PCR was performed using gene-specific primers against *DIP1*, *DHN1*, and *RAB21*. **b** *OsWRKY11*-kd and non-transgenic (WT) control plants were subjected to drought stress. Samples were collected 6 DAT. RT-PCR was performed using gene-specific primers against *OsWRKY11*, *RAB21*, *DHN1*, and *DIP1*. The transcript level of *OsACTIN* was used as an internal control. These experiments were repeated more than twice

The expression of drought-responsive genes in transgenic plants over-expressing *OsWRKY11* has not been previously investigated (Wu et al. 2009). We therefore analyzed expression of drought-responsive genes, including dehydrins such as *DIP1*, *DHN1*, and *RAB21* (Fig. 5a). These three dehydrins were constitutively expressed in transgenic *OsWRKY11*-ox plants. By contrast, induction of dehydrins following drought stress was compromised in *OsWRKY11*-kd lines (Fig. 5b). All these results indicate that *OsWRKY11* positively regulates the expression of drought-responsive genes.

### *OsWRKY11* trans-activates the *RAB21* promoter by direct binding in vivo

We demonstrated that *Os*WRKY11 was a transcription activator and drought-responsive genes were up-regulated in *OsWRKY11*-ox lines but these effects were compromised in *OsWRKY11*-kd lines. To examine whether *Os*WRKY11 directly regulated drought-responsive genes as it did defense-related genes, we performed transient assays of promoter activity in *N. benthamiana*.

**Fig. 6:**
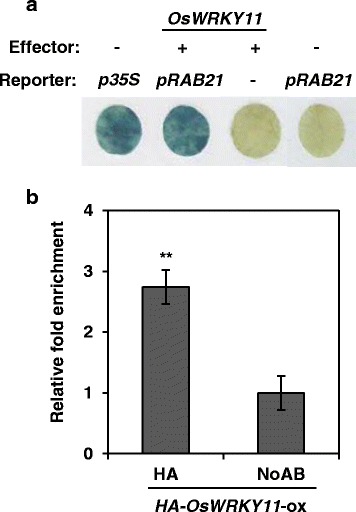
*Os*WRKY11 directly binds to and activates the *RAB21* promoter. **a** *Nicotiana benthamiana* leaf discs were infiltrated with *Agrobacterium* carrying *pRAB21::GFP-GUS* alone or with a mixture of *Agrobacterium* carrying *35S::OsWRKY11* and *pRAB21::GFP-GUS*. Promoter activity in each sample was visualized 2 days post-infiltration using β-glucuronidase (GUS) activity staining. **b** Transgenic rice leaves expressing *35S*::*HA*-*OsWRKY11* were used for chromatin immunoprecipitation with anti-HA antibody. ChIP-PCRs were performed on genomic DNA fragments using promoter-specific primers against *RAB21* (Table S1). To normalize qPCR values, the value obtained from the sample with pre-immune serum (no antibody) was divided by the value obtained from the sample with 10% input of each sheared chromatin sample and arbitrarily set at 1. The value obtained from *Os*WRKY11 binding to the promoter was expressed as a relative ratio of the pre-immune serum sample. Asterisks indicate significant differences between treatments from the no-antibody control sample (**: *P* < 0.01; *: *P* < 0.05)

A promoter-reporter construct containing the *RAB21* promoter upstream of a GFP-GUS fusion gene (*pRAB21::GFP-GUS*) was generated and transformed into *Agrobacterium*. *Agrobacterium-*mediated transient assays were performed in *N. benthamiana* leaves. Trans-activational activity of *Os*WRKY11 at the *RAB21* promoter was assessed using GUS staining (Fig. 6a; Additional file 1: Figure S2). GUS activity was stronger in leaves co-infiltrated with *pRAB21::GFP-GUS* and *35S::OsWRKY11* than in leaves infiltrated with either *pRAB21::GFP-GUS* or *35S::OsWRKY11*. This suggested that *Os*WRKY11 trans-activated the *RAB21* promoter *in planta*.

In vivo binding of *Os*WRKY11 to the *RAB21* promoter was also assessed in transgenic plants over-expressing HA-OsWRKY11 using ChIP with anti-HA antibody (Fig. 6b; Additional file 1: Figure S4). The signal obtained by PCR with primers binding sites near the W-box or WLE1 of the *RAB21* promoter was higher in IP samples treated with anti-HA antibody than in no-antibody samples, suggesting that *Os*WRKY11 binds directly to the promoter .

### *OsWRKY11* protein is controlled by the Ubiquitin-Proteasome system

**Fig. 7:**
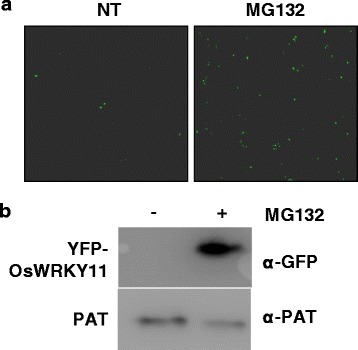
Degradation of *Os*WRKY11 protein by the UPS. **a** Rice (*Oryza sativa*) protoplasts were transformed with 35S::*OsWRKY11-YFP*. After 4 h, protoplasts were treated with 100 μM MG132 and buffer for 1 day. Fluorescence was observed using a confocal laser scanning microscope. Scale bars: 30 μm. NT: non-treated control. **b** Immunoblot analysis of total protoplast proteins in the absence (−) or presence (+) of 100 μM MG132. Blots were probed with green fluorescent protein (GFP) antibody. The PAT antibody was used as a control for transformation efficiency

To prevent untimely activation of immune response transcription factors such as OsWRKY6 and OsWRKY45 are often controlled by the ubiquitin-proteasome system (UPS) (Choi et al., 2015; Matsushita et al., 2013). To examine whether *Os*WRKY11 was regulated by the UPS, we monitored the effect of the proteasome inhibitor MG132 on *Os*WRKY11 protein accumulation. GFP florescence was monitored in protoplasts transformed with 35S::YFP-OsWRKY11 (Fig. 7a). GFP florescence was stronger in protoplasts transfected with 35S::YFP-OsWRKY11 in the presence of MG132. This is suggesting that the level of *Os*WRKY11 may be controlled by the UPS. YFP-*Os*WRKY11 protein was detected by the immuno-blot analysis with a GFP antibody (Fig. 7b). *Os*WRKY11 protein levels were higher in the MG132-treated sample than in the mock-treated sample, while PAT protein levels were similar in both samples, again suggesting that the UPS controls levels of *Os*WRKY11 protein.

## Discussion

A number of studies show that WRKY TFs are involved in the integration of signaling pathways in biotic and abiotic stress responses negatively or positively (reviewed in Sharma et al. 2013; Kissoudis et al. 2014; Takatsuji 2017). WRKY TFs involved in positive cross-talk are thought to be good candidates for proteins conferring tolerance to abiotic and biotic stresses; however, there has been no consideration of the potential uses of these TFs in improving tolerance to abiotic and biotic stresses. This may be because the positive effects of those TFs were reported by independent research groups. Here, we report the involvement of *Os*WRKY11 in positive cross-talk between abiotic and biotic stress responses.

*OsWRKY11* is induced by heat and drought stresses, and over-expression of *OsWRKY11* under the control of the HSP101 promoter enhances drought tolerance (Shiroto et al. 2004; Wu et al. 2009). In addition, *OsWRKY11* expression is induced by pathogens such as *M. grisea* and *Xoo* (Ryu et al. 2006). There are, however, no further reports on the function of *Os*WRKY11 in pathogen-mediated defense signaling beyond these studies of *OsWRKY11* expression. The expression pattern of *OsWRKY11* prompted us to consider it a good candidate for playing a role in positive cross-talk between abiotic and biotic stresses.

*Os*WRKY11 is a member of group IIc (Xie et al. 2005), which contains 18 *Os*WRKY proteins (Berri et al. 2009). An *Arabidopsis* protein in this group, *At*WRKY75, is involved in Pi stress as well as pathogen defense (Devaiah et al. 2007; Encinas-Villarejo et al. 2009; Choi et al. 2014). *Os*WRKY31 (*Os*WRKY23, according to CGSNL) confers enhanced resistance to *Pseudomonas syringae* when expressed heterologously in *Arabidopsis* (Jing et al. 2009; Berri et al. 2009). To assess the involvement of *Os*WRKY11 in pathogen-mediated defense signaling transgenic lines over- or under-expressing *OsWRKY11* were therefore generated. *OsWRKY11*-ox lines displayed reduced susceptibility to bacterial leaf blight caused by a bacterial pathogen *Xoo*, while this reduced susceptibility was compromised in *OsWRKY11*-kd lines.

Defense-associated genes, such as *PR10*, *Betv1*, and *CHIT2*, were constitutively expressed in *OsWRKY11*-ox lines, while their expression was compromised in *OsWRKY11*-kd lines, indicating that *Os*WRKY11 was a positive regulator of expression of defense-associated genes (Peng et al. 2008). There are 125 WRKY TFs in rice (Rice WRKY Working Group, 2012), and, of these, *Os*WRKY03 (12 by CGSNL), 6, 13, 31 (23, by CGSNL), 45, 53, 62, 71, and 89 (104, by CGSNL) are known to be positive regulators of defense-associated genes (Liu et al. 2005; Li et al. 2006; Choi et al. 2015; Chujo et al. 2007; Liu et al. 2007; Shimono et al. 2007; Qiu et al. 2007; Zhang et al. 2008; Chujo et al. 2009; Hwang et al. 2011). Out of the *Os*WRKY proteins, only the functions of *Os*WRKY6, *Os*WRKY13, *Os*WRKY45, and OsWRKY62 (Fukushima et al., 2016) have been thoroughly investigated using kd or knock-out strategies. Our analysis of *OsWRKY11*-kd lines confirmed that *Os*WRKY11 functioned as a positive regulator in the expression of defense-associated genes, thereby conferring disease resistance, in a similar manner to the other WRKY factors described above.

A number of studies show that WRKY TFs act as transcriptional activators or repressors in pathogen-mediated defense signaling. Our trans-activation assay in yeast demonstrated that *Os*WRKY11 functioned as a transcriptional activator. The protein contains the consensus coactivator motif LXLL (L: leucine; X: any amino acid) (Xie et al. 2005). This motif may contribute to transcriptional activation of reporter genes. TFs mostly localize to the nucleus, either by themselves or with help from other protein(s). OsWRKY11 was localized in nuclei. The putative NLS may contribute to nuclear localization of *Os*WRKY11 (Xie et al. 2005). In addition, we used ChIP and transient assays of promoter activity to confirm that *Os*WRKY11 could bind directly to the *CHIT2* promoter and induce transcriptional activation in vivo. We demonstrated that OsWRKY11 trans-activated and bound around the W box or WLE1 of the *CHIT2* promoter in vivo. There was no report yet that OsWRKY TF directly regulates the expression of the chitinase in vivo in our knowledge. Previously we reported that OsWRKY6 directly regulates *OsPR10a* promoter (Choi et al. 2015).

It was previously reported that *Os*WRKY11 is induced by heat and enhances drought tolerance (Wu et al. 2009). However, this study did not investigate the downstream target genes of OsWRKY11 in terms of drought tolerance. We demonstrated that over-expressing *OsWRKY11* under the control of the 35S promoter resulted in enhanced tolerance of drought, as previously reported (Wu et al. 2009). Moreover, we observed constitutive expression of drought-responsive genes in *OsWRKY11*-ox lines and that induction of drought-responsive genes, such as *RAB21*, *DHN1*, and *DIP1*, was compromised in *OsWRKY11-*kd lines during drought stress. Our ChIP and promoter transient assays showed that direct binding of *Os*WRKY11 to the *RAB21* promoter led to transcriptional activation in vivo. Taken together, our data indicate that *Os*WRKY11 is a positive regulator of drought-responsive genes, and thereby enhances drought tolerance. Each TF positively or negatively regulates a subset of stress-related genes. In line with this concept, *Os*WRKY11 positively regulates drought-responsive genes, including several dehydrin genes.

Positive regulators of rice immunity, such as *Os*NPR1, *Os*WRKY6, and *Os*WRKY45, are often subject to control by the UPS to prevent untimely activation of the immune response (Choi et al. 2015; Liu et al. 2017; Matsushita et al. 2013). *Os*WRKY11 was also controlled by the UPS. Plant defense responses to abiotic and biotic stresses theoretically impart a cost of reduced growth and reproduction (recently reviewed in Karasov et al. 2017; Takatsuji 2017), and thus plants are likely to develop various means to minimize growth/defense trade-offs. Degradation of immune proteins by the UPS is considered one such strategy, and therefore degradation of *Os*WRKY11 by the UPS may underlie the beneficial phenotypes for aspects of plant performance observed in transgenic lines over-expressing *Os*WRKY11, such as plant height and yield.

## Conclusions

Plants are normally exposed to abiotic and biotic stresses in their natural environment. Drought stress and diseases are major causes of reduced crop yields. To overcome both stresses efficiently, plants must establish elaborate cross-talk between signaling pathways at various levels. We found that *Os*WRKY11 was a positive regulator of plant defense responses to drought and pathogens, including the bacterium, *Xoo*. Many transgenic approaches have been performed to increase biotic and abiotic stress tolerance (Kissoudis et al. 2014). Transgenic rice cultivars with improved drought tolerance and resistance to two severe diseases can increase and stabilize crop yields in stressful environments. Our data indicated that *OsWRKY11* is a good candidate for improving the yield of transgenic crops. Identification of upstream factor(s) regulating *Os*WRKY11 is required to elucidate *Os*WRKY11-mediated cross-talk between the abiotic and biotic stress response pathways.

## Methods

### Plant materials and treatments

Transgenic rice seedlings (*Oryza sativa* L. ssp. *japonica* cv. Nipponbare) were grown in a greenhouse for 3 weeks for RT-PCR analysis, and for 6 weeks for studies of disease severity. *Xoo* KACC10331, a compatible strain to the Nipponbare cultivar, were grown in peptone-sucrose-agar medium (10 g peptone, 10 g sucrose, 1 g sodium-glutamate, and 15 g agar per liter) at 28 °C for 2 days and resuspended in 10 mM MgCl_2_ to an OD_600_ of 0.5.

Transgenic rice seedlings (Nipponbare cultivar) were inoculated with *Xoo* using a toothpick at approximately 10 mm intervals. Samples were taken at the times indicated and stored at −80 °C prior to RNA isolation and RT-PCR analysis. For RT-PCR analysis of gene expression under drought conditions, 6-week-old seedlings were drought-treated, and samples were collected approximately 6 days after treatment (dat).

### Vector construction and generation of transgenic rice plants

A full-length *OsWRKY11* cDNA clone was obtained from cDNA reverse transcribed from total RNA extracted from rice leaves infected with *Xoo* by PCR (Additional file 1: Table S1). The PCR product was amplified using primers OsWRKY11-F and R, and cloned into pDONR221 using BP clonase (Invitrogen, Carlsbad, CA) to make an entry clone. This entry clone was confirmed by sequencing.

The 35S::OsWRKY11 and 35S::HA-OsWRKY11 constructs were produced using an LR reaction between the entry clone and the destination vectors pB2GW7 (Karimi et al. 2002) and pEarleygate201 (Earley et al. 2006), respectively. The *35S::OsWRKY11* RNAi construct was produced using an LR reaction between the entry clone pDONR201-OsWRKY11-RNAi and pB7GWIW2 (II) (Karimi et al. 2002).

The three constructs were introduced into rice using *Agrobacterium*-mediated transformation as previously described (Kim et al., 2009) with minor modifications. *Agrobacterium* LBA4404 carrying each construct was used to infect rice callus generated from *Oryza sativa* cv. Nipponbare. Transgenic lines were generated by transforming rice with the 35S::*OsWRKY11-*ox construct or the 35S::*OsWRKY11* RNAi construct.

### RT-PCR analysis

Leaf samples were ground to powder in liquid nitrogen, and total RNA was isolated for RT-PCR, as described (Hwang et al. 2008). RT-PCR analysis using M-MLV RTase (Promega, Madison, WI, USA) was performed using 1 μg total RNA according to the manufacturer’s instructions. Subsequent PCR was performed using 25–30 cycles and gene-specific primers (Additional file 1: Table S1). *OsActin* (XM469569) (Hwang et al. 2008) primers were used as a loading control. All gel images are generated by a program (Quantity one-4.5.2) in gel documentation system (Bio Rad Gel Doc XR system US170–8170) and trimmed around corresponding band for each gene in size.

### Disease assays

For *Xoo* inoculation, the bacteria were grown on peptone-sucrose-agar plates at 28 °C for 2 days and resuspended in 10 mM MgCl_2_ to an OD_600_ of 0.5 (Hwang et al. 2011). *OsWRKY11*-ox lines (T1) and *OsWRKY11* RNAi lines (T1) were challenged with a compatible strain of *Xoo* strain KXO85 (KACC10331) using the leaf-clip method (Kauffman et al. 1973). Lesion lengths in each plant were measured at 14 days post-inoculation (dpi). Disease severity was expressed as a percentage of lesion length (cm)/leaf length for individual plants (Tao et al. 2009).

### Promoter transient expression assay

A 1.5 kb region of the *RAB21* (Os11g26790) promoter were amplified using PCR with specific primers (Additional file 1: Table S1) and introduced into an entry vector, pENTR/d-TOPO (Invitrogen, Carlsbad, CA, USA). *pRAB21::GFP-GUS* constructs were made by an LR reaction between the entry clone containing the *pRAB21* promoter and the destination vector pBGWFS7 (Karimi et al. 2002). A 2.0 kb region of the *CHITINASE 2* (*CHIT2*; Os04g41620) promoter were amplified using PCR with specific primers in Additional file 1: Table S1 and used for entry vector. *pCHIT2::GFP-GUS* constructs were made by an LR reaction between the entry clone containing the *pCHIT2* promoter and the destination vector pBGWFS7. Transient expression assays in *Nicotiana benthamiana* were performed using the protocol reported previously (Li, X. 2011). *N. benthamiana* was infiltrated with *Agrobacterium* carrying *pCHIT2::GFP-GUS* and *pRAB21::GFP-GUS* constructs alone, or with a mixture of *Agrobacterium* carrying *35S::OsWRKY11* and either *pCHIT2::GFP-GUS* or *pRAB21::GFP-GUS*. Infiltrated leaves were collected 2 days post-infiltration, and promoter activities in each sample were visualized using β-glucuronidase (GUS) activity staining. Leaf disks were immersed in a solution of 1 mM 5-bromo-4-chloro-3-indolyl-b-glucuronic acid in 100 mM sodium phosphate pH 7.0 plus 0.1% Triton X-100 and incubated at 37 °C before clearing in 70% ethanol.

### Chromatin immunoprecipitation (ChIP)

Chromatin immunoprecipitation (ChIP) assays were generally followed as previously described (Haring et al. 2007). Transgenic rice leaves over-expressing *35S*::*HA*-*OsWRKY11* were harvested and fixed with formaldehyde under a vacuum. The chromatin was sheared by sonication and mixed with anti-HA antibody (Abcam, Cambridge, UK). Protein A agarose (Thermo Scientific, Rockford, IL, USA) was added to precipitate the DNA fragments.

ChIP-PCRs were performed using promoter-specific primers against *CHIT2* and *RAB21* (Additional file 1: Table S1, see Supporting Information). qPCR values were normalized with the value obtained from the sample with pre-immune serum (no antibody) divided by the value obtained from the sample containing 10% input of each sheared chromatin was arbitrarily set at 1, and subsequent values resulting from *Os*WRKY11 binding to the promoter were expressed as relative ratios of the pre-immune serum sample. Chromatin samples precipitated with pre-immune serum were used as negative controls in all ChIP-qPCR reactions. Similar results were obtained in three independent experiments.

### Protein accumulation assay for OsWRKY11 in rice protoplasts

Protoplasts were prepared from dark-grown rice seedlings for 10 days. PEG-mediated transformation was performed as previously described (Bart et al. 2006). The vector *35S::YFP-OsWRKY11* was introduced into rice protoplasts as described previously. After 4 h, 100 μM MG132, a proteasome inhibitor was added to the protoplasts and incubated for 1 day. Green fluorescent protein (GFP) fluorescence was examined under a confocal laser scanning microscope (Olympus FV300; Olympus, Germany).

Proteins extracts were prepared in ice-cold extraction buffer [150 mM NaCl, 50 mM Tris-Cl (pH 7.5), 1 mM EDTA, 0.5% NP-40, 1 mM PMSF, 50 μM MG132, and one protease inhibitor cocktail tablet per 10 ml; Roche, Mannheim, Germany]. Proteins were separated using 12% sodium dodecyl sulfate–polyacrylamide gel electrophoresis (SDS-PAGE). Immunoblot analysis was performed with GFP antibody (Abcam; code: ab6556). Immunoblot analysis with phosphinothricin acetyltransferase (PAT) antibody (Abcam; code: ab1791) was used as a loading control.

## Additional file

Additional file 1: Table S1. Primers used in this study. **Figure S1.** Subcellular localization and transcriptional activity of *Os*WRKY11. **Figure S2.** Schematic maps of constructs used for transient assays of promoter activity. **Figure S3.** Activation of the *CHIT 2* promoter by *Os*WRKY11 in rice. **Figure S4.** W-box and W-box-like element 1 (WLE1) depicted in *CHIT2* and *RAB21* promoter. **Figure S5.** Drought response assays for plants over (ox)- or under-expressing (RNAi-knock-down; kd) *OsWRKY11*. Supplementary experimental procedures. (DOCX 130 kb)
