# Supplementary material for: Rice WRKY11 Plays a Role in Pathogen Defense and Drought Tolerance
Source: Rice (N Y). 2018 Jan 12;11:5. doi: 10.1186/s12284-018-0199-0 (PMC5766476; doi:10.1186/s12284-018-0199-0)
Supplement: Additional file 1: Table S1. — Primers used in this study. Figure S1. Subcellular localization and transcriptional activity of OsWRKY11. Figure S2. Schematic maps of constructs used for transient assays of promoter activity. Figure S3. Activation of the CHIT 2 promoter by OsWRKY11 in rice. Figure S4. W-box and W-box-like element 1 (WLE1) depicted in CHIT2 and RAB21 promoter. Figure S5. Drought response assays for plants over (ox)- or under-expressing (RNAi-knock-down; kd) OsWRKY11. Supplementary experimental procedures. (DOCX 130 kb) [file 12284_2018_199_MOESM1_ESM.docx]

| **Table S1**. Primers used in this study | | | |
| --- | --- | --- | --- |
| **Gene** | **Accession no.** | **Sequence (5' to 3')** | **Experiment** |
| *OsWRKY11* | Os01g43650 | F: 5’-**AAAAAGCAGGCTCG**ATGTCTTCTGGAGGAGGA-3’  R: 5’-**AGAAAGCTGGGTA**TGGGTTGCTGCTGGGCATT-3’ | Full gene cloning |
|  |  | F: 5’-**AAAAAGCAGGCTC**GATCACCACGTACGAAGGGC-3’  R: 5’-**AGAAAGCTGGGT**AGTCAGTGAAGTGATGCTGC-3’ | RNAi vector construction |
| *CHIT2* | Os04g41620 | F: 5’-**CACC**ATGATATGGCTGTGGGTCCCA-3’  R: 5’-GCTGCAGCTTAGCACGTACATG-3’ | Promoter cloning |
| *RAB21* | Os11g26790 | F: 5’-**CACC**CTGACTGCACTGCAGAGA-3’  R: 5’-GAGATCGAGGTGTTCTT-3’ | Promoter cloning |
| *OsWRKY11* | Os01g43650 | F: 5’-AGCCCAGAGAGAAAGCTGAG-3’  R: 5’-TATGGGCTGTTCTTGACTGC-3’ | RT-PCR |
| *OsActin* | XM469569 | F: 5’-TCCATCTTGGCATCTCTCAG-3’  R: 5’-GTACCCGCATCAGGCATCTG-3’ | RT-PCR |
| *PR10* | BAD03969 | F: 5’-CGCAGCTCACATTATCAAGTCAGA-3’  R: 5’-GAAGCAGCAATACGGAGATGGATG-3’ | RT-PCR |
| *Betv1* | NP001066995 | F: 5’-GCAGGGAGCGTATACAAGACCAA-3’  R: 5’-CACGCCACAGTAACATGACCACAA-3’ | RT-PCR |
| *CHIT2* | Os04g41620 | F: 5’-CAAGAGCAACAAACAGTGGC-3’  R: 5’-CGCCCTAAGATAGAGTAACATCG-3’ | RT-PCR |
| *RAB21* | AK073487 | F: 5’-TGCATCTTCTACGACCACATGTTCGACT-3’  R: 5’-CTGGTTTGAGCATGACTATCCCGTTCAG-3’ | RT-PCR |
| *DIP1* | AY587109 | F: 5’-GGCTTTAGCTGAATCCATCG-3’  R: 5’-CTCCTTGAGCCCCTTCTTCT-3’ | RT-PCR |
| *DHN1* | AY786415 | F: 5’-TGCTCCTGCCACTACTCCGG-3’  R: 5’-TGGTCTCACACACGCCCCAA-3’ | RT-PCR |
| *CHIT2* | Os04g41620 | F: 5’-GGCAGCCTCTCGACTTTCGG-3’  R: 5’-TGAAGGTTTATGGTGGCCGTC-3’ | ChIP |
| *RAB21* | Os11g26790 | F: 5’-ACGCATCCTCTCCTATCTCC-3’  R: 5’-TTATAAGCCGGAGGAGGCGA-3’ | ChIP |
| AttB1  AttB2 |  | 5’-GGGGACAAGTTTGTACAAAAAAGCAGGCT-3’  5’-GGGGACCACTTTGTACAAGAAAGCTGGGT-3’ | Vector construction |

The overhang sequences in these primers required for vector construction are marked in bold.

**a**


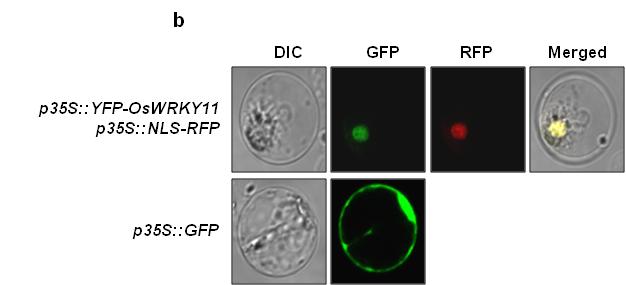


**b**

*
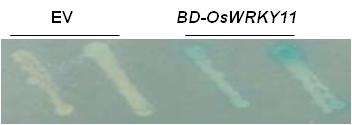
*

Fig. S1. Subcellular localization and transcriptional activity of OsWRKY11. (a) YFP::*Os*WRKY11 fusion protein expression was driven by the 35S promoter. *YFP::OsWRKY11* and *NLS::RFP* were introduced into rice protoplasts using PEG-mediated transformation. As a control, a parent vector was transformed into separate cells. These experiments were repeated twice with similar results. (b) BD*-OsWRKY11* was constructed using Gateway cloning into the destination vector pDEST32. Yeast cells were transformed with *BD-OsWRKY11* or a parent vector. Color changes were monitored on plates overlaid with X-α-Gal.

Fig. S2. Schematic maps of constructs used for transient assays of promoter activity. GFP: green fluorescent protein; GUS: β-glucuronidase; T35S: terminator of 35S.


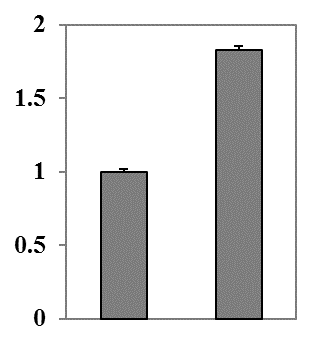


**Relative luciferase activity**

**Reporter:**

**Effector:**

**a**

**b**

**Effector:**

***p35S***

***OsWRKY11***

***T35S***

**Reporter:**

***pCHIT2***

***LUC***

***T35S***

**-**

**+**

**+**

**+**

******

Fig. S3. Activation of the *chit2* promoter by *Os*WRKY11 in rice. Protoplasts from wild-type plants were transformed with *pCHIT2::LUC* plus either the empty vector (*pB2GW7*) or *35S::OsWKRY11*. Transformed protoplasts were analyzed 24 h after PEG-mediated transformation to quantify relative LUC activity. Protoplasts were co-transformed with 35S-renilla(r)_LUC to normalize the transformation efficiency; the fLUC/rLUC ratio obtained from protoplasts transformed with *pCHIT2::LUC* and the empty vector was set to an arbitrary value of 1.

Fig. S4. W-box and W-box-like element 1 (WLE1) depicted in *Chitinase 2* (*CHIT2*) and Rab21 promoter. Arrows indicate position of primers used for ChIP-qPCR.

*
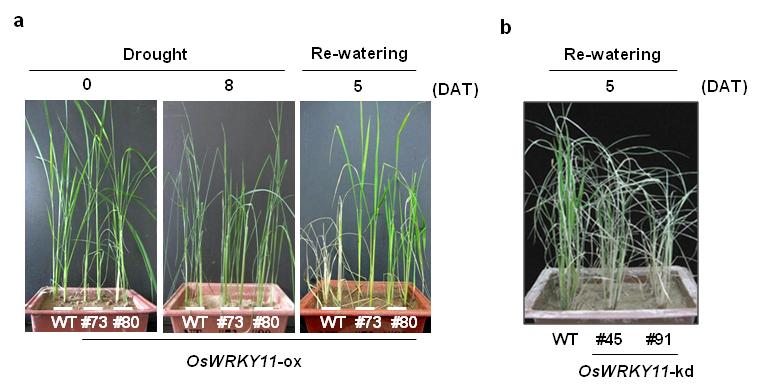
*

Fig. S5. Drought response assays for plants over (ox)- or under-expressing (RNAi-knock-down; kd) *OsWRKY11*. (a) *OsWRKY11*-ox and non-transgenic (WT) control plants were subjected to drought stress. Photographs were taken 0 (left-hand panel) and 8 days (middle panel) after treatment (DAT). Plants were re-watered 10 DAT, and a photograph was taken after 5 days of re-watering (right-hand panel). (b) *OsWRKY11*-kd and non-transgenic (WT) control plants were subjected to drought stress. Plants were re-watered 10 DAT, and a photograph was taken after 5 days of re-watering

**Supplementary experimental procedures**

*Trans-activation assay*

To examine the transcriptional activity of OsWRKY11, an expression construct for GAL4-binding domain::OsWRKY11 (BD::OsWRKY11) protein was produced by an LR reaction between an entry clone containing *OsWRKY11* and the GAL4 BD vector (pDEST32). Yeast cells (AH109; Invitrogen) were transformed with the protein construct. Yeast cells carrying either BD::OsWRKY11 or empty vector were grown on selective medium plates without leucine, over-laid with 2 mg/mL X-α-Gal to monitor color changes of the cells.

*Subcellular localization*

The YFP::OsWRKY11 construct was produced by an LR reaction between the OsWRKY11 entry clone and pEarleyGate 104 (Earley et al., 2006). The constructs were introduced into rice protoplasts using PEG-mediated transformation (Bart et al., 2006). Protoplasts were viewed with a confocal microscope (Olympus FV300 CLSM, Japan) under bright field, a YFP filter, or a RFP filter 24–48 h after transformation.

*Promoter transient expression assay*

A 2 kb region of the *chitinase 2* (*CHIT2*) promoter was isolated using PCR with promoter-specific primers and introduced into an entry vector, pENTR/d-TOPO (Invitrogen, Carlsbad, CA). The *pCHIT2::LUC* construct was made by LR reaction between the entry clone containing the promoter and a promoter destination vector (Hwang et al., 2008). Protoplasts from wild-type plants were transformed with *pCHIT2-LUC* alone or co-transformed *pCHIT2-LUC* and 35S-OsWRKY11. Luciferase activities were measured using the dual luciferase assay system (Promega, Madison, WI, USA), according to the manufacturer’s instructions. *35::RLUC* (*Renilla* luciferase; Hwang et al., 2008) was used to measure the transformation efficiency between samples, and firefly luciferase activity was normalized to *Renilla* luciferase activity.

*Drought tolerance assays*

For the drought tolerance assays, plants were grown in pots under greenhouse conditions for 6 weeks. Water was then withheld for 10 days to allow the soil to dry until plants displayed wilting. Potted plants were re-watered after 10 days in drought conditions. Photographs were taken on the indicated days.

**Reference for supplementary material**

Hwang SH, Lee IA, Yie SW, Hwang DJ **(**2008) Identification of an *OsPR10a* promoter region responsive to salicylic acid. Planta 227:1141-1150
